# Quality indicators for opioid stewardship interventions in hospital inpatient and emergency departments: a systematic review

**DOI:** 10.1097/PR9.0000000000001284

**Published:** 2025-06-18

**Authors:** Chelsea Dutkiewicz, Katelyn Jauregui, Shania Liu, Racha Dabliz, Asad E. Patanwala, Jonathan Penm

**Affiliations:** a The University of Sydney, Faculty of Medicine and Health, School of Pharmacy, Camperdown, Sydney, NSW, Australia; b Department of Pharmacy, Campbelltown Hospital, Campbelltown, NSW, Australia; c Department of Pharmacy, Royal Prince Alfred Hospital, Camperdown, NSW, Australia; d Department of Pharmacy, Prince of Wales Hospital, Randwick, NSW, Australia

**Keywords:** Opioid, Prescribing, Hospitals, Inpatient, Emergency department, Pain management

## Abstract

A systematic review was conducted to identify existing quality indicators for opioid stewardship in hospital inpatient and emergency departments. Five databases (MEDLINE, Embase, CINAHL, CENTRAL, and Scopus) and grey literature were searched up to April 2022 to identify articles containing quality indicators for opioid stewardship in the hospital setting. Quality indicators were classified into (1) structure, (2) process, and (3) outcome according to Donabedian model and further analysed as subgroups. Risk of bias assessment was performed by 2 independent reviewers. In total, 4621 articles were identified after duplicates were removed, of which 20 full-text articles were screened for eligibility and 8 articles included in the review. There were 81 quality indicators extracted, of which 49 were unique quality indicators. Analysis produced 25% (12/49) structure, 61% (30/49) process, and 14% (7/49) outcome quality indicators. These quality indicators may be used to developed future quality standards, although further study is needed to improve patient data collection for outcome quality indicators to allow for more holistic evaluation of opioid stewardship programs.

## Abstract 2

Supplemental Digital Content is Available in the Text.

This systematic review identified 81 quality indicators for opioid stewardship, which can be applied to develop future standards.

## 1. Introduction

Excessive opioid use is becoming a global issue, with a World Health Organisation report finding 70% of deaths attributable to drug use worldwide related to opioid use.^40^ Rising opioid prescription rates have been observed in many developed countries and identified as a driving factor of this “opioid epidemic,”^19,33,58,59^ with a U.K. study finding a 34% increase in opioid prescription rates between 1998 and 2016, equal to a 127% increase in opioid consumption when corrected for strength of opioid prescribed.^14^ Multiple studies have also highlighted the role of hospital prescribing practices in increasing opioid prescription rates.^6,7,9,18,48,53,54^ Prominent issues include the large number of opioids supplied on discharge, long-term use of opioids after elective surgeries, and inappropriate prescribing to opioid-naïve patients.^6,7,9,18,48,53,54^ For example, a recent meta-analysis found that on average 61% of postoperative opioids provided on discharge remained unused, which translated to 27 tablets leftover per person.^48^ Hence, the rising opioid prescription rates observed globally may be driven by inappropriate opioid prescribing practices in the hospital setting.

Multiple interventions have been implemented to reform opioid use in the hospital setting in response to this crisis.^2,4,11,27,35,37,43,44,60^ For example, academic detailing and education for health professionals, pain monitoring to determine appropriate treatment, as well as patient education have all led to improved opioid prescribing as well as improved patient and clinical outcomes.^35^ A relatively novel intervention is the implementation of an opioid stewardship program in hospitals. Opioid stewardship programs use evidence-based guidelines and practices to oversee the responsible use of opioids with the aim to optimize patient outcomes by minimizing opioid-related harms and maximising clinical benefits.^4,11,20,27,35,50^ This governance structure includes aspects such as pain assessment, risk assessment for opioid-related harm, and reviewing opioid utilization before and after surgical procedures.^11,27^ However, the best measure to evaluate the performance of opioid stewardship programs in clinical care settings is unknown.^4^

As such, quality indicators have been developed to measure the performance of opioid stewardship programs and improve their implementation in hospitals. Quality indicators are a widely accepted tool in health care to measure practice performance and change in quality-of-care provided.^32^ Consensus of quality indicators allows for comparison between sites, and their review is effective at improving quality of healthcare processes and patient outcomes.^10,15^ For example, a review of quality indicator performance resulted in increased clinician responsiveness in areas such as policy and procedure changes, educational programs, new job roles, and equipment changes.^10^ However, no consensus yet exists on the quality indicators for opioid stewardship programs because of their novelty in hospital settings. Therefore, the objectives of this study were to identify all existing quality indicators for opioid stewardship programs in inpatient and emergency departments to guide future quality standards. Moreover, identification of these quality indicators would allow for future classification of their importance, improving opioid stewardship programs and thus appropriate opioid utilisation.

## 2. Methods

This review was conducted and reported according to the *Preferred Reporting Items for Systematic Reviews and Meta-Analyses* (PRISMA) statement.^41^ The protocol for this systematic review was registered on PROSPERO (ID: CRD42022321371).

### 2.1. Study selection

Inclusion criteria included full-text peer-reviewed literature or those published by international regulatory bodies and commissions with quality indicators for opioid stewardship in the hospital inpatient or emergency department setting. Articles were excluded if they were published in a language other than English, were only published as conference abstracts, or were editorials, literature reviews, commentaries, or did not include a quality indicator.

The study selection process involved exporting the studies from the electronic databases into Endnote X9 and removing duplicate studies.^39^ Two reviewers screened the remaining studies based on title and abstract to remove irrelevant articles. The full texts of the selected studies were reviewed against the exclusion criteria to confirm eligibility. In addition, the reference lists of the included studies were checked for further eligible studies not discovered in the electronic search. The databases and grey literature were searched on April 27, 2022, to identify potentially relevant sources.

### 2.2. Search strategy

Two approaches were used to identify all relevant studies and guidelines. First, 5 electronic databases were systemically searched, including MEDLINE, Embase, and CENTRAL via Ovid, CINAHL via EBSCOhost, as well as Scopus via Elsevier. The search terms used were based on 4 key concepts: (1) opioids, (2) hospital—inpatient and emergency settings, (3) appropriate use, and (4) quality indicators (see Appendix 1, http://links.lww.com/PR9/A315 for the full search strategy). No limitation was placed on the searches, and dates ranged from database inception until April 27, 2022.

Second, a grey literature search was performed to identify additional quality indicators not indexed in the databases above (search terms used: opioid OR pain, quality indicators OR standards). This included a search of international regulatory bodies and commissions until April 27 2022, including:

(1) Accreditation Canada.^8^
(2) Agency for Healthcare and Research Quality.^45^
(3) Australian Commission on Safety and Quality in Healthcare.^24^
(4) Grey Matters.^38^
(5) Institute for Healthcare Improvement.^28^
(6) National Health Services.^49^
(7) National Institute for Health and Care Excellence.^21^
(8) The Canadian Patient Safety Institute.^29^
(9) The Joint Commission.^13^

### 2.3. Data extraction

Two independent reviewers extracted data from the full texts of relevant articles. Data included author, country, year, methodology, participants, funding of the articles, and a description of the quality indicators. Quality indicators that contained the same concept were consolidated into one to identify conceptually unique quality indicators. Where the overall methodology of quality indicator conception was unclear, all mentioned techniques were extracted. Where funding was unclear for grey literature sources, the organisational body was assumed responsible.

### 2.4. Data analysis

Quality indicators were classified into (1) structure, (2) process, or (3) outcome based on Donabedian model and further analysed as subgroups.^16^ Applying Donabedian model to this study, structure relates to the framework or attributes of the setting in which the opioid stewardship program was implemented, process relates to the actions performed within the program, and outcome relates to the effect of the program on patient outcomes. Subgroups within these headings were determined retrospectively by 2 reviewers to allow for further comparison. Subgroups were decided based on a similar core concept seen in 2 or more quality indicators, where applicable, and any disagreement between the 2 reviewers was resolved by consulting a third reviewer.

Structure quality indicators refer to those concerned with the environment in which care was delivered, including materials or human resources within the hospital setting.^16^ Subgroups for structure quality indicators included: “Documentation,” “Hospital policy,” and “Resources.” Process quality indicators refer to the actions of the health professionals, such as opioid prescribing or monitoring patients.^16^ Process quality indicators were further divided into subgroups including: “Risk assessment,” “Prescribing Decisions,” “Monitoring,” and “Discharge.” Outcome quality indicators refer to how the patient has been affected from the care provided, such as pain intensity or length of hospital stay.^16^ These were divided into the subgroups: “Pain,” “Quality of life,” and “Mortality.”

Moreover, the data were analysed to identify any current gaps in quality indicators for opioid stewardship or sections that need development. Classifications or subgroups with proportionally fewer quality indicators were considered a “gap” or needing development. Distribution of quality indicator classifications within and between articles was compared with authorship and other extracted data from the articles such as participants, or funding, to identify potential causes for difference.

### 2.5. Quality assessment

Two independent reviewers performed risk of bias assessments for all included studies using a consensus-based method using the *Consensus-based Standards for the Selection of Health Measurement Instruments* (*COSMIN*) *Risk of Bias tool* for studies that used a Delphi method.^52^ Included grey literature documents were critically appraised using a modified version of the *Authority, Accuracy, Coverage, Objectivity, Date, and Significance* (*AACODS*) *checklist*.^57^

## 3. Results

**Figure 1.**
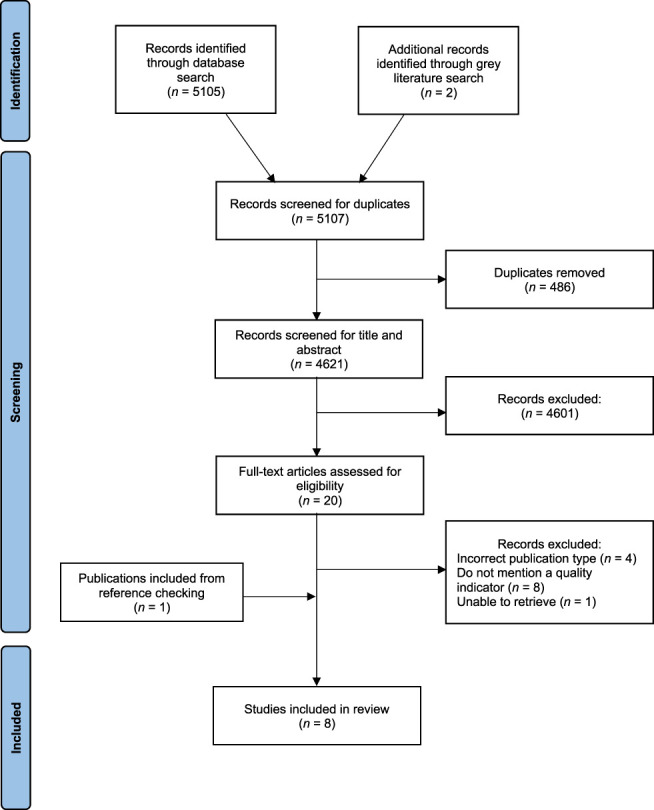
PRISMA flowchart of the literature search.

Initially, 5,105 articles were obtained from the database search, and 2 additional articles were identified from the grey literature search. In total, 486 duplicate records were removed from this sample. The remaining 4,621 articles were screened based on title and abstracts. Twenty articles were assessed for eligibility based on their full text, of which 13 articles were excluded for the following reasons: wrong publication type (n = 4), did not mention a quality indicator (n = 8), or the full text was unable to be retrieved (n *=* 1). One additional relevant article was identified from the references of included articles. Eight articles met all inclusion criteria and were included in the review (Fig. 1).

### 3.1. Study characteristics

Of the 8 articles, half were based in the United States (n = 4),^12,46,47,55^ 2 in the United Kingdom,^22,56^ 1 based in Canada,^3^ and 1 in Australia.^11^ A modified Delphi or eDelphi method was used in 75% (6/8) of articles and participants commonly included organisational groups or committees made of physicians, pharmacists, nurses, as well as various experts in pain and addiction medicine.^3,22,46,47,55,56^ Three articles had quality indicators specific to special patient groups, including paediatrics,^22^ geriatrics,^55^ and patients with established opioid use disorder, respectively.^47^

A total of 81 quality indicators were extracted from the included articles, of which 49 were unique quality indicators (Table 1). Analysis of the unique quality indicators based on the Donabedian model produced 24% (12/49) structure, 61% (30/49) process, and 14% (7/49) outcome quality indicators (Tables 2 and 3). All articles included at least one process quality indicator. Four articles did not include a structure quality indicator,^3,22,55,56^ and 4 articles did not include an outcome quality indicator.^11,22,55,56^

**Table 1:** Data extracted from included articles, including quality indicators classified by Donabedian model (structure, process, and outcome).

Author (y), country	Methodology	Participant/s*	Quality indicators			Funding
			Donabedian's model	Subgroup	Description	
Allen et al. (2020), Canada	Modified Delphi method	1. Definition's Outcomes Group (including physicians, pharmacists, methodologists) 2. National Opioid Use Guideline Group	Structure	*N/A*	No applicable data	Michael G. DeGroote National Pain Centre, McMaster University, Hamilton
			Process	*Screening*	Proportion of patients who have their risk of addiction assessed with a screening tool prior to initiating therapy for chronic noncancer pain	
					Frequency of methods used by health care providers to assess risk of addiction, eg, validated scales vs informal assessment	
				*Monitoring*	Proportion of patients on opioid therapy for chronic noncancer pain who are monitored for aberrant drug-related behaviours by physicians and pharmacies	
					Proportion of physicians and pharmacists who routinely monitor for aberrant drug-related behaviour in their patients on opioid therapy for chronic noncancer pain	
					Proportion of physicians and pharmacists who routinely monitor for aberrant drug-related behaviour in their patients on opioid therapy using prescription monitoring program patients information, where available	
			Outcome	*Quality of life*	Change in scores on validated quality of life scales and function scales for patients with chronic noncancer pain treated with opioids (eg, SF-36)	
Fox et al. (2016), United Kingdom	eDelphi method	1. Royal College of Paediatrics and Child Health & Neonatal and Paediatric Pharmacists Group 2. Contacts from the National Institute of Healthcare Research Programme 3. General paediatricians, paediatric pharmacists, and paediatric clinical pharmacologists (United Kingdom)	Structure	*N/A*	No applicable data	University Hospitals, Southampton
			Process		Prescribing of incorrect or inequivalent morphine (opioid) dose via multiple routes	
			Outcome	*N/A*	No applicable data	
Rizk et al. (2019), United States	Modified Delphi method	1. Houston Methodist Health System, Research Institute, and University of Houston (including physicians, pharmacists, nurses, administrators, managers, and outcome researchers) 2. Houston Methodist System Pain Management Committee	Structure	*Documentation*	Proportion of hospitalised patients who have documentation of patient defined pain goals	Vigilanz corporation
					Proportion of hospitalised patients with opioid orders who have a standardised documentation of the pain management plan (eg, use of approved order sets)	
					Proportion of patients who have documentation of patient defined function goals	
			Process	*Prescribing decisions*	Proportion of hospitalised patients who received naloxone	
					Proportion of hospitalised patients who are opioid naïve and have long acting or extended-release opioid orders (methadone, patches, extended-release formulations)	
					Proportion of hospitalised patients with concurrent administrations of high doses of opioids and at least 1 medication from the following classes: benzodiazepines, barbiturates, sedative hypnotics, GABA analogues, or muscle relaxants	
					Proportion of hospitalized patients receiving IV opioid pushes	
					Average dose of MME administered per day	
					Proportion of patients with opioid doses ≥90 MME/day	
					Proportion of patients with opioid doses ≥50 MME/day	
				*Monitoring*	Proportion of patients who have multiple prn opioid orders with duplicate prn indication	
				*Discharge*	Proportion of opioid discharge prescriptions that exceed 7 d of treatment	
					Proportion of patients with opioid discharge prescriptions given in the ED that exceed 3–5 d	
					Proportion of patients discharged on opioids who receive discharge education on opioid purpose, adverse effects, monitoring, secure storage/disposal, and alternatives	
					Proportion of patients discharged from the hospital with opioid discharge prescriptions	
					Proportion of discharged patients with opioid discharge prescription of ≥50 MME/day	
			Outcome	*Pain*	Proportion of hospital days with 1 or more severe pain rating score	
Samuels et al. (2019), United States	Modified Delphi method	1. Experts in quality measurement from American College of Emergency Physicians (ACEPs) Clinical Emergency Data Registry 2. Researchers in emergency and addiction medicine 3. Representatives from federal agencies (National Institute on Drug Abuse, Centers for Medicare & Medicaid Services)	Structure	*Hospital policy*	“Safe prescribing” ED policies	None
					ED naloxone distribution policy	
					Structured screening and diagnostic questionnaires	
				*Resources*	Availability of nonopioid pain management	
					Prescription Drug Monitoring Programs-Electronic Medical Record (PDMP-EMR) integration	
			Process	*Prescribing decisions*	Trial of nonopioid analgesics before opioid initiation when indicated	
					Median days opioids prescribed	
					Frequency of benzodiazepine and opioid coprescribing	
					Proportion of ED OUD patients with initiated medication for OUD	
					Proportion of ED OUD patients with prescribed medication for OUD	
					Median MME/day per ED visit	
				*Discharge*	Patient education about opioid safe storage and disposal on discharge	
					Proportion of ED OUD patients provided with overdose prevention and response patient education	
					Proportion of ED OUD patients discharged with/prescribed naloxone	
					Proportion of ED OUD patients linked to outpatient OUD treatment	
					Proportion of ED OUD patients counselled by health promotion advocates, counsellors, or social workers	
			Outcome	*Quality of life*	Adverse events after ED discharge after receiving new opioids	
					ED revisitation for analgesia-associated adverse medication events	
					Risk-adjusted 30-d repeated ED visit for nonfatal opioid overdose	
				*Mortality*	Risk-adjusted in-hospital mortality for overdose	
Terrell et al. (2009), United States	Modified Delphi method (ACOVE quality indicator approach for initial list)	1. Society for Academic Emergency Medicine (SAEM) Geriatric Task Force 2. Audience at 2007 SAEM Annual Meeting 3. Audience at 2008 workshop for American Geriatrics Society annual meeting	Structure	*N/A*	No applicable data	Award from the American Geriatrics Society (AGS) (supported by John A. Hartford Foundation)
			Process	*Prescribing decisions*	If an older adult receives analgesic medication while in the ED, then meperidine should be avoided	
				*Discharge*	If an older adult receives an opioid analgesia prescription upon discharge from the ED, then a bowel regimen should also be provided (or the provider should document why a bowel regimen was not given)	
			Outcome	*N/A*	No applicable data	
Thomas et al. (2013), United Kingdom	eDelphi method	1. Recruited from National Electronic Prescribing Conference and personal contacts of research team (including pharmacists, clinical pharmacologists, physicians)	Structure	*N/A*	No applicable data	Research grant from National Institute for Health Research
			Process	*Prescribing decisions*	Regular opioids prescribed without concurrent use of laxatives	
					Prescribing of incorrect or inequivalent morphine (opioid) dose via multiple routes	
			Outcome	*N/A*	No applicable data	
Joint Commission (2017), United States	1. Literature Review, Public Field Review 2. Technical advisory panel discussion to identify innovative, high-quality, safe initiatives in the field of pain assessment and management 3. Learning visits at hospitals	1. Technical advisory panel representing members of leading healthcare organisations 2. Standards Review Panel	Structure	*Hospital policy*	The hospital has a leader or leadership team that is responsible for pain management and safe opioid prescribing and develops and monitors performance improvement activities	Joint Commission Resources Inc
					The hospital provides information to staff and licensed independent practitioners on available services for consultation and referral of patients with complex pain management needs	
					The hospital identifies opioid treatment programs that can be used for patient referrals	
					The hospital has defined criteria to screen, assess, and reassess pain that are consistent with the patient's age, condition, and ability to understand	
				*Resources*	The hospital provides nonpharmacologic pain treatment modalities	
					The hospital provides staff and licensed independent practitioners with educational resources and programs to improve pain assessment, pain management, and the safe use of opioid medications based on the identified needs of its patient population	
					The hospital facilitates practitioner and pharmacist access to the prescription Drug Monitoring program databases	
					Hospital leadership works with its clinical staff to identify and acquire the equipment needed to monitor patients who are at high risk for adverse outcomes from opioid treatment	
				*Documentation*	The hospital collects data on pain assessment and pain management including types of interventions and effectiveness	
					The hospital analyses data collected on pain assessment and pain management to identify areas that need change to increase safety and quality for patients	
			Process	*Screening*	The hospital screens patients for pain during emergency department visits and at the time of admission	
				*Prescribing decisions*	The hospital develops a pain treatment plan based on evidence-based practices and the patient's clinical condition, past medical history, and pain management goals	
					The hospital involves patients in the pain management treatment planning process through: developing realistic expectations and measurable goals that are understood by the patient for the degree, duration, and reduction of pain; discussing the objectives used to evaluate treatment progress; providing education on pain management, treatment options, and safe use of opioid and nonopioid medications when prescribed	
					The medical staff is actively involved in pain assessment, pain management, and safe opioid prescribing through participating in the establishment of protocols and quality metrics, and reviewing performance improvement data	
				*Monitoring*	The hospital monitors patients identified as being high risk for adverse outcomes related to opioid treatment	
					The hospital reassesses and responds to the patient's pain through the evaluation and documentation of response(s) to pain intervention(s); progress toward pain management goals including functional ability; side effects of treatment; and risk factors for adverse events caused by treatment	
				*Discharge*	The hospital educates the patient and family on discharge plans related to pain management including pain management plan of care; side effects of pain management treatment; activities of daily living, including the home environment, that might exacerbate pain or reduce effectiveness of the pain management plan of care, as well as strategies to address these issues; safe use, storage, and disposal of opioids when prescribed	
			Outcome	*Pain*	The hospital treats the patient's pain or refers the patient for treatment	
Australian Commission on Safety and Quality in Healthcare (2022), Australia	1. Review of existing Australian and International QIs 2. Prioritisation, review, and refinement of the QIs in consultation with the topic working group and MSOC	1. Opioid Regulatory Advisory Group (established by the Therapeutic Goods Administration)	Structure	*Hospital policy*	Evidence of a locally approved policy that defines the process for managing admitted patients identified as being at increased risk of opioid-related harm who are prescribed an opioid analgesic, including: process for identifying patients who may be at risk of opioid-related harm, local pathways for managing patients identified at increased risk of opioid-related harm, systems to inform patients why they are being referred to a pathway and the plan for their ongoing clinical management, process for clinicians to refer patients to appropriate support services and escalate care to specialist services, process to ensure clinicians are competent in the use of the pathway, process to assess adherence to the pathway	Australian Government
					Evidence of a locally approved policy to support the transfer of care of patients who separate from hospital with a supply or prescription of opioid analgesics. This should specify the: organisation's opioid analgesic weaning and cessation protocol, process for referral to specialist services (if required), required documentation to be provided to the patient or carer, required clinical handover documentation to be provided to the general practitioner, process to ensure the workforce is competent in the use of the policy, process to assess adherence to the policy	
			Process	*Screening*	Proportion of patients who received opioid analgesics who had pain and functional assessments prior to being prescribed opioid analgesics and the outcomes of the assessments documented in their medical record	
				*Prescribing decisions*	Proportion of patients who were newly prescribed opioid analgesics who were coprescribed CNS depressant medicines while in hospital	
					Proportion of admitted patients who received opioid analgesics who were administered naloxone for respiratory depression	
					Proportion of admitted patients who received opioid analgesics who also received prophylactic laxatives to prevent opioid constipation	
				*Monitoring*	Proportion of patients separated from hospital with a supply or prescription of opioid analgesics where a Real Time Prescription Monitoring program or prescription shopping program was checked prior to separation	
					Proportion of admitted patients who received opioid analgesics where the intended number of days of treatment was documented in their medical record	
				*Discharge*	Proportion of patients separated from hospital with a supply or prescription of opioid analgesics who also received a supply or prescription of paracetamol and nonsteroidal anti-inflammatory medicines	
					Proportion of opioid-naïve surgical patients separated from hospital with a supply or prescription of opioid analgesics where the supply or prescription was for a modified-release formulation	
					Proportion of overnight admitted patients separated from hospital with a supply or prescription of opioid analgesics that exceeded the opioid analgesic inpatient dose given during the 24 h prior to separation	
					Proportion of patients separated from the ED with a supply or prescription of opioid analgesics where the supply exceeded 3 d of treatment	
					Proportion of patients separated from hospital with a supply or prescription of opioid analgesics: a) where the supply or prescription exceeded 7 d of treatment b) whose medication management plan was given to the patient or carer on separation c) whose medication management plan was sent to the general practitioner on separation	
			Outcome	*N/A*	No applicable data	

* Where multiple participants are listed, they were involved in separate iterative reviews of the Delphi method.

CNS, central nervous system; ED, emergency department; IV, intravenous; MME, morphine milligram equivalents; N/A, not applicable; OUD, opioid use disorder; SF-36, 36-Item Short Form Survey Instrument.

**Table 2:** Unique quality indicators organised into subgroups of Donabedian model (structure, process, and outcome).

Group	Subgroup	Quality indicator(s)
Structure	Documentation	1. Proportion of hospitalised patients who have documentation of patient defined pain and function goals^39^ 2. Proportion of hospitalised patients with opioid orders who have a standardised documentation of the pain management plan^39^ 3. The hospital analyses data collected on pain assessment and management to identify types of interventions and effectiveness, as well as areas needing change^38^
	Hospital policy	1. The hospital has defined criteria to screen, assess, and reassess pain that are consistent with the patient's age, condition, and ability to understand^38^ 2. The hospital has a 'safe prescribing' emergency department policy and defines the process for managing admitted patients identified at increased risk of opioid-related harm^21,40^ 3. The hospital provides information to staff, licensed independent practitioners, and other staff involved in patient referrals on available services for patients with complex pain management needs or requiring opioid treatment programs^38^ 4. The hospital has a leader or leadership team responsible for pain management and safe opioid prescribing that develops and monitors performance improvement activities^38^ 5. The hospital has a policy to support the transfer of care of patients who separate from hospital with a supply or prescription of opioid analgesics^21^
	Resources	1. The hospital provides nonopioid pain treatment^38,40^ 2. The hospital provides adequate access to Prescription Drug Monitoring Programs^38,40^ 3. The hospital provides staff and licensed independent practitioners with educational resources and programs to improve treatment of pain and safe opioid use based on the needs of its patient population^38^ 4. Hospital leadership works with its clinical staff to identify and acquire the equipment needed to monitor patients who are at high risk for adverse outcomes from opioid treatment^38^
Process	Screening	1. Proportion of patients who have their risk of addiction assessed with a screening tool prior to being prescribed opioid analgesics^42^ 2. Proportion of patients who had pain and functional assessments prior to being prescribed opioid analgesics^21,38^
	Prescribing Decisions	1. Prescribing of incorrect or inequivalent morphine (opioid) dose through multiple routes^43,44^ 2. Proportion of hospitalized patients receiving IV opioid pushes^40^ 3. Proportion of patients with opioid doses ≥50 MME/day and ≥90 MME/day^39^ 4. Median days opioids prescribed^40^ 5. Proportion of patients trialled on nonopioid analgesics before opioid analgesics are initiated^40^ 6. Proportion of hospitalised patients who are opioid naïve and have long acting or extended-release opioid orders (methadone, patches, extended-release formulations)^39^ 7. Proportion of patients who were prescribed opioid analgesics and coprescribed CNS depressants^21,39,40^ 8. Proportion of hospitalised patients who received naloxone^21,39^ 9. Proportion of admitted patients who received opioid analgesics who also received prophylactic laxatives to prevent opioid constipation^21,44^ 10. Proportion of emergency department opioid use disorder patients with prescribed medication for opioid use disorder^40^ 11. The medical staff involve the patient in the pain management treatment planning process, tailor it to the patient's clinical condition, and base it on evidence-based practices^37^ 12. Proportion of older adults requiring analgesic medication receiving pethidine^41^
	Monitoring	1. Proportion of patients who have multiple prn opioid orders with duplicate prn indication^39^ 2. Proportion of patients separated from hospital with a supply or prescription for opioid analgesics who were monitored for aberrant drug-related behaviours or checked using prescription monitoring/shopping program^21,42^ 3. The hospital monitors patients identified as being high risk for adverse outcomes related to opioid treatment^38^ 4. The hospital reassesses and responds to the patient's pain through the evaluation and documentation of response(s) to pain intervention(s); progress toward pain management goals including functional ability; side effects of treatment; and risk factors for adverse events caused by treatment^38^ 5. Proportion of admitted patients who received opioid analgesics where the intended number of days of treatment was documented in their medical record^21^
	Discharge	1. Proportion of patients separated from the ED with a supply or prescription of opioid analgesics where the supply exceeded 3 days of treatment^21,39^ 2. Proportion of opioid discharge prescriptions that exceed 7 d of treatment^21,39^ 3. Proportion of discharged patients with opioid discharge prescription of ≥50 MME/day^39^ 4. Proportion of overnight admitted patients separated from hospital with a supply or prescription of opioid analgesics that exceeded the opioid analgesic inpatient dose given during the 24 h prior to separation^21^ 5. Proportion of opioid-naïve surgical patients separated from hospital with a supply or prescription of opioid analgesics where the supply or prescription was for a modified-release formulation^21^ 6. Proportion of patients discharged on opioids who receive discharge education on opioid purpose, adverse effects, monitoring, secure storage/disposal, and alternatives^38–40^ 7. Proportion of patients separated from hospital with a supply or prescription of opioid analgesics whose medication management plan was given and explained to the patient or carer on separation^21,38^ 8. Proportion of patients separated from hospital with a supply or prescription of opioid analgesics whose medication management plan was sent to the general practitioner on separation^21^ 9. Proportion of older adults with an opioid analgesic prescription upon discharge from the ED provided with a bowel regimen^41^ 10. Proportion of ED OUD patients discharged with or prescribed naloxone or other overdose prevention^40^ 11. Proportion of patients separated from hospital with a supply or prescription of opioid analgesics who also received a supply or prescription of paracetamol and nonsteroidal anti-inflammatory medicines^21^
Outcome	Pain	1. Proportion of hospital days with one or more severe pain rating score^39^ 2. The hospital treats the patient's pain or refers the patient for treatment^38^
	Quality of life	1. Change in scores on validated quality of life scales and function scales for patients with chronic noncancer pain treated with opioids (eg, SF-36)^42^ 2. Adverse events after ED discharge after receiving new opioids^40^ 3. ED revisitation for analgesia-associated adverse medication events^40^ 4. Risk-adjusted 30-d repeated ED visit for nonfatal opioid overdose^40^
	Mortality	1. Risk-adjusted in-hospital mortality for overdose^40^

CNS, central nervous system; ED, emergency department; IV, intravenous; MME, morphine milligram equivalents; OUD, opioid use disorder; SF-36, 36-Item Short Form Survey Instrument.

**Table 3:** Summarised unique quality indicators in subgroups of Donabedian model.

Group	Subgroup	Summary of quality Indicator(s)
Structure	Documentation	Pain and function goals specified Standardised pain management plan documented Data used to assess effectiveness of interventions
	Hospital policy	Defined criteria to screen, assess and reassess pain based on patient characteristics “Safe prescribing” policy in the emergency department Information provided to staff on available services for complex pain management and opioid treatment programs Pain management leadership team Transfer of care policy for opioid analgesic supply
	Resources	Nonopioid pain treatment and access to prescription drug monitoring programs Educational resources and programs for safe opioid use Access to equipment for monitoring adverse opioid outcomes
Process	Screening	Risk of addiction, pain and functional assessments assessed prior to opioid analgesic prescription
	Prescribing Decisions	Incorrect, inequivalent, or high opioid dose received, and IV opioid pushes given Median days of opioids prescribed Nonopioid analgesics trialled before opioid analgesics initiated Opioid-naïve patients given extended-release opioids CNS depressants coprescribed, naloxone or prophylactic laxatives received Community opioid use disorder prescriptions continued on admission to the emergency department Patient involved in pain management treatment planning Older patients receiving pethidine
	Monitoring	Multiple prn opioid orders for duplicate indication Patients monitored for aberrant drug-related behaviours given opioid supply or prescription on discharge Patients at high risk for adverse outcome monitored Continually reassess and adjust patient's pain management considering patient response, functional ability, side effects and risk factors Intended duration of opioid treatment documented in medical record
	Discharge	Greater than 3 or 7 d supply or prescription of opioids given Greater than 50 MME/day of opioids prescribed Opioid-naïve patients given extended release opioids Supply or prescription greater than inpatient use 24 h prior to discharge given to overnight admitted patients Education or medication management plan given to patient or carer Medication management plan sent to GP. Older patients counselled on appropriate bowel regimen ED OUD given naloxone or other overdose prevention Patient given simple analgesia in addition to opioid analgesics
Outcome	Pain	Severe pain rating on ≥1 d, and pain treated or referred for treatment
	Quality of life	Change in scores on validated quality of life scales and function scales for patients with chronic noncancer pain treated with opioids Adverse events after ED discharge after receiving new opioids ED revisitation for analgesia-associated adverse medication events Risk-adjusted 30-d repeated ED visit for nonfatal opioid overdose
	Mortality	Risk-adjusted in-hospital mortality for overdose

CNS, central nervous system; ED, Emergency Department; GP, general practitioner; IV, intravenous; MME, morphine milligram equivalents; OUD, opioid use disorder.

#### 3.1.1. Structure quality indicators

Structure quality indicators were addressed by half of the included articles (4/8) and resulted in 12 unique quality indicators (12/49).^11,12,46,47^ The subgroup “Documentation” contained 3 quality indicators (3/12) related to pain management plans and documentation of pain and function goals. The subgroup “Hospital policy” contained 5 quality indicators (5/12) related to set screening and assessment criteria for patient pain, “safe prescribing policies” such as identifying patients at high risk of opioid-related harm, as well as staff hierarchy and leadership teams. The subgroup “Resources” contained 4 quality indicators (4/12) related to nonopioid or nonpharmacological pain treatment alternatives, and staff access to prescription drug monitoring programs, educational resources, or other equipment deemed necessary for monitoring patients at high-risk of opioid-related harm. Quality indicators relating to the number of personnel or methods of peer review were absent from the included articles. In addition, the Joint Commission report was the only article to consider human resources in their structure quality indicators.^12^

#### 3.1.2. Process quality indicators

Process quality indicators included 30 unique quality indicators (30/49), thus being the largest category of Donabedian model considered in the included articles. The subgroup “Screening” contained 2 quality indicators (2/30) related to screening for risk of opioid use disorder as well as pain and functional assessments before prescribing opioids. The subgroup “Prescribing Decisions” contained the most quality indicators (12/30) and was considered by most included articles (7/8).^11,12,22,46,47,55,56^ These quality indicators related to administration of opioids for route and dose measured in morphine milligram equivalents, duration of opioid prescriptions, coprescriptions with other central nervous system depressant drugs, as well as prophylactic laxative use and naloxone administration. Process quality indicators related to special patient groups considered the use of pethidine in geriatric patients, opioid-naïve patients receiving extended-release opioid orders, and the use of prophylactic laxatives in both paediatric and geriatric cases. The subgroup “Monitoring” contained 5 quality indicators (5/30) related to multiple “prn” (give as needed) opioid orders, monitoring for aberrant drug-related behaviour or risk of opioid-related harm, as well as documenting patient response and length of opioid treatment. The subgroup “Discharge” contained the second highest number of quality indicators, containing 11 quality indicators (11/30) related to characteristics of opioid prescriptions on discharge such as length or strength of treatment, as well as other practitioner activities on discharge including patient education, provision of overdose prevention, and coprescription with nonopioid analgesics. This subgroup was also considered by most of the included articles (5/8).^11,12,46,47,55^

#### 3.1.3. Outcome quality indicators

Seven unique outcome quality indicators (7/49) were identified from this review. The subgroup “Pain” contained 2 quality indicators (2/7) related to patient pain rating score and whether it was treated. The subgroup “Quality of life” contained 4 quality indicators (4/7) related to patient quality of life and function score, adverse events experienced, and emergency department revisitation. The subgroup “Mortality” contained one quality indicator (1/7) related to in-hospital mortality for opioid overdose. These outcomes considered health status at the patient level rather than the population level. Other quality indicator concepts not addressed included improvement in patient knowledge, changes in patient behaviour, and patient satisfaction with care.

### 3.2. Quality assessment

Risk of bias assessment was performed for all included studies. Of the 6 articles (6/8) that had a consensus-based methodology, all were considered “Inadequate” or as having a high risk of bias as the quality indicators were not determined independently from other participants (Appendix 2, http://links.lww.com/PR9/A315).^3,22,46,47,55,56^ The 2 grey literature articles (2/8) included were assessed as having a low risk of bias (Appendix 3, http://links.lww.com/PR9/A315).^11,12^

## 4. Discussion

The results of this review demonstrate that quality indicator metrics for opioid stewardship in inpatient and emergency departments primarily focus on the actions of the health professional. This reflects current opioid prescribing guidelines, which predominantly focus on safer opioid prescribing practices to improve opioid utilisation.^19,30,31^ Safer opioid prescribing practices in the hospital aim to avoid issues such as large number of opioids supplied on discharge, long-term use of opioids after elective surgeries, and inappropriate prescribing of long acting opioids to opioid-naïve patients.^34,36^ Quality indicators in this study address these “problem” areas of opioid prescribing highlighted in the literature, such as trialling nonopioid analgesics before opioid analgesics,^18^ avoiding long-acting opioid orders in opioid-naïve patients,^18,33^ and limiting the quantity of opioids supplied on discharge.^7,18,48,53,54^ Such quality indicator metrics should be considered in opioid stewardship programs, as liberal opioid prescribing practices have often been implicated in cases of opioid-related harm.^14,25,51,58^ For example, a study by Brat et al.^6^ found that each additional week of opioid prescription was associated with a 20% increase in opioid misuse and harm. Avoiding liberal discharge prescriptions was especially prominent in quality indicators addressing prescribing in the emergency department, perhaps indicative of studies finding initiation of opioid prescriptions for minor, acute pain after emergency department visits as a “gateway” to recurrent opioid use, and thus opioid-related harm.^26^ Indeed, quality indicators on discharge from emergency departments in this study were “stricter” than those for inpatient settings, with smaller quantities of opioids to be supplied on discharge. Hence, the composition of quality indicators in this review demonstrates the importance of assessing opioid prescribing practices to evaluate opioid stewardship programs.

The results of this study additionally highlight the role of the organisation in effective opioid stewardship, through the secondary focus on structural quality indicators. This reflects the importance of structural support to reform the actions of the health professional, a core concept of Donabedian model, whereby good structure begets good processes, and good processes beget good outcomes.^17^ For example, a study by Penm et al.^42^ elucidated the impact of structural support on prescribing decisions of emergency physicians, whereby physician income was related to patient satisfaction scores. Physicians were motivated to prescribe higher doses of opioids to ensure high patient satisfaction, regardless of the increased risks involved.^42^ Therefore, reforming structural factors is an important consideration to assess opioid stewardship programs in hospital, due to its effect on the actions of the health professional.

Moreover, despite the goal of opioid stewardship programs to improve opioid utilisation for the sake of the patient, there was a noted paucity of outcome quality indicators when assessing opioid stewardship programs. This composition of quality indicators is similar to a study on the responsible use of medicine, which found that 94% of quality indicators were focused on process and only 3% on patient outcomes.^23^ Furthermore, this lack of assessment of patient outcomes may be due to feasibility issues in collecting data for these metrics, as patient-led parameters, such as patient satisfaction, may not easily be obtained through readily available data sources such as the medical record and may lack a standardised form of measurement to allow for succinct comparison.^5,17,23^ Indeed, issues of “credibility” and “feasibility” were important considerations of many of the included studies for the conception of their quality indicators. Therefore, current evaluation of opioid stewardship programs may lack focus on direct patient-related outcomes due to feasibility issues. Further study is needed to devise more feasible ways of recording patient data for quality improvement efforts.

### 4.1. Strengths and limitations

The main strength of this review includes the comprehensive search strategy, which considered multiple databases in addition to grey literature to capture a broader subset of the current literature. This ensured that relevant unpublished, organisational quality standards were also considered. However, owing to the novelty of opioid stewardship programs in hospital, there was a limited amount of literature available. As such, the search strategy included any quality indicator related to opioid prescribing, resulting in the inclusion of papers that considered prescribing quality indicators generally, which may affect the specificity of the results to opioid stewardship programs. Moreover, another limitation of this review was only including articles published in English. As such, all included studies were set in English-speaking countries, which may limit the applicability of these findings to other settings. However, overuse of opioids in the hospital systems seems to be more common in Western Countries, which primarily publish in English.^1^ In addition, most included articles were assessed as having a high risk of bias.^3,22,46,47,55,56^ This is ultimately due to the nature of the methodology being consensus-based, and thus, the results were not obtained independently. Finally, our study did not find a minimum set of quality indicators to be used within certain hospital areas, as this was outside the scope of our review.

### 4.2. Implications for policy and practice

The results of this study identify existing quality indicators for institutions to construct a quality standard for opioid stewardship programs, which considers all aspects of Donabedian model: process, structure, and outcome quality indicators. Based on this review, the actions of the health professional are the most important factor to assess the effectiveness of opioid stewardship programs. Future quality standards should consider the screening and monitoring of patients, prescribing decisions, as well as discharge prescriptions to ensure adequate evaluation of opioid utilisation. As seen in this review, stricter limitations on discharge prescriptions for the emergency department may be required, due to the link between opioid initiation in emergency departments and persistent opioid use.^7,18,26^ Moreover, organisational factors should be considered when assessing opioid stewardship programs due to its cascading effect on the actions of the health professionals. Outcome quality indicators may be covered in less depth, due to current feasibility issues in collecting these data; however, future studies should address these issues to allow for inclusion of more outcome quality indicators and thus a more holistic evaluation of opioid stewardship programs.

## 5. Conclusion

This review identified 49 unique quality indicators for opioid stewardship programs for hospital inpatient and emergency department settings, which may be used to guide future quality standards. Evaluation of opioid stewardship programs should consider quality indicators covering processes of care, followed by structural organisation and patient outcomes. Future studies should assess the feasibility of such indicators to ensure holistic and effective evaluation of opioid stewardship programs.

## Disclosures

The authors have no conflict of interest to declare.

## Appendix A. Supplemental digital content

Supplemental digital content associated with this article can be found online at http://links.lww.com/PR9/A315.

## Supplementary Material

SUPPLEMENTARY MATERIAL

## References

[1] Al-Samawy S Varughese N Vaillancourt R Wang XY Penm J. A global survey on opioid stewardship practices in hospitals: a cross-sectional pilot study. Pharmacy 2021;9:122. 34287334 10.3390/pharmacy9030122 PMC8293457

[2] Alexandridis AA McCort A Ringwalt CL Sachdeva N Sanford C Marshall SW Mack K Dasgupta N. A statewide evaluation of seven strategies to reduce opioid overdose in North Carolina. Inj Prev 2018;24:48–54. 28835443 10.1136/injuryprev-2017-042396 PMC5795575

[3] Allen M Sproule B MacDougall P Furlan A Murphy L Borg Debono V Buckley N. Identifying appropriate outcomes to help evaluate the impact of the Canadian guideline for safe and effective use of opioids for non-cancer pain. BMC Anesthesiol 2020;20:6–9. 31910806 10.1186/s12871-020-0930-4 PMC6945645

[4] Awadalla R Gnjidic D Patanwala A Sakiris M Penm J. The effectiveness of stewardship interventions to reduce the prescribing of extended-release opioids for acute pain: a systematic review. Pain Med 2020;21:2401–11. 32488237 10.1093/pm/pnaa139

[5] Black N Jenkinson C. Measuring patients' experiences and outcomes. BMJ 2009;339:b2495. 19574317 10.1136/bmj.b2495

[6] Brat GA Agniel D Beam A Yorkgitis B Bicket M Homer M Fox KP Knecht DB McMahill-Walraven CN Palmer N Kohane I. Postsurgical prescriptions for opioid naive patients and association with overdose and misuse: retrospective cohort study. BMJ 2018;360:j5790. 29343479 10.1136/bmj.j5790 PMC5769574

[7] Calcaterra SL Yamashita TE Min S-J Keniston A Frank JW Binswanger IA. Opioid prescribing at hospital discharge contributes to chronic opioid use. J Gen Int Med 2016;31:478–85. 10.1007/s11606-015-3539-4 PMC4835366 26553336

[8] Accreditation Canada. Ottawa: Accreditation Canada. Available at: https://accreditation.ca/. Accessed April 27, 2022.

[9] TASMAN Collaborative. Patterns of opioid use after surgical discharge: a multicentre, prospective cohort study in 25 countries. Anaesthesia. 2024;79:924–36. 38721718 10.1111/anae.16297

[10] Collopy BT. Clinical indicators in accreditation: an effective stimulus to improve patient care. Int J Qual Health Care 2000;12:211–6. 10894192 10.1093/intqhc/12.3.211

[11] Australian Commission on Safety and Quality in Health Care. Opioid analgesic stewardship in acute pain clinical care standard. Sydney: ACSQHC; 2022.

[12] The Joint Commission. Pain assessment and management standards for hospitals: The joint commission. Oakbrook Terrace: The Joint Commission; 2017:7.

[13] The Joint Commission. Oakbrook Terrace: The Joint Commission. Available at: https://www.jointcommission.org/. Accessed April 27, 2022.

[14] Curtis HJ Croker R Walker AJ Richards GC Quinlan J Goldacre B. Opioid prescribing trends and geographical variation in England, 1998–2018: a retrospective database study. Lancet Psychiatry 2019;6:140–50. 30580987 10.1016/S2215-0366(18)30471-1

[15] De Vos M Graafmans W Kooistra M Meijboom B Van Der Voort P Westert G. Using quality indicators to improve hospital care: a review of the literature. Int J Qual Health Care 2009;21:119–29. 19155288 10.1093/intqhc/mzn059

[16] Donabedian A. Explorations in quality assessment and monitoring: the definition of quality and approaches to its assessment. Ann Arbor: Health Administration Press; 1980.

[17] Donabedian A. The quality of care: how can it be assessed? JAMA 1988;260:1743–8. 3045356 10.1001/jama.260.12.1743

[18] Donohue JM Kennedy JN Seymour CW Girard TD Lo-Ciganic W-H Kim CH Marroquin OC Moyo P Chang C-CH Angus DC. Patterns of opioid administration among opioid-naive inpatients and associations with postdischarge opioid use: a cohort study. Ann Int Med 2019;171:81–90. 31207646 10.7326/M18-2864 PMC6815349

[19] Dowell D Haegerich TM Chou R. CDC guideline for prescribing opioids for chronic pain—United states, 2016. JAMA 2016;315:1624–45. 26977696 10.1001/jama.2016.1464 PMC6390846

[20] Dutkiewicz C Liu S Patanwala A McLachlan AJ Stevens J Khor KE Bugeja B Begley D Fong I Jauregui K Penm J. Clinicians' perspective of the opioid analgesic stewardship in acute pain clinical care standard. Health Pol Technol 2024;13:100936.

[21] National Institute for Health and Care Excellence (NICE). London: NICE. Available at: https://www.nice.org.uk/. Accessed April 27, 2022.

[22] Fox A Pontefract S Brown D Portlock J Coleman J. Developing consensus on hospital prescribing indicators of potential harm for infants and children. Br J Clin Pharmacol 2016;82:451–60. 27038331 10.1111/bcp.12954 PMC4972161

[23] Fujita K Moles RJ Chen TF. Quality indicators for responsible use of medicines: a systematic review. BMJ Open 2018;8:e020437. 10.1136/bmjopen-2017-020437 PMC6082479 30012782

[24] Australian Commission on Safety and Quality in Health Care (ACSQHC). Sydney: ACSQHC. Available at: https://www.safetyandquality.gov.au/. Accessed April 27, 2022.

[25] Hedegaard HWM Minino AM. Drug overdose deaths in the United States 1999–2016. National Center for Health Statistics, Centers for Disease Control and Prevention. Available at: wwwcdcgov/nchs/products/databriefs/db294html. Accessed April 3, 2018. 27996932

[26] Hoppe JA Kim H Heard K. Association of emergency department opioid initiation with recurrent opioid use. Ann Emerg Med 2015;65:493–9.e4. 25534654 10.1016/j.annemergmed.2014.11.015

[27] Hyland SJ Brockhaus KK Vincent WR Spence NZ Lucki MM Howkins MJ Cleary RK. Perioperative pain management and opioid stewardship: a practical guide. Healthcare (Basel) 2021;9:333. 33809571 10.3390/healthcare9030333 PMC8001960

[28] Institute for Healthcare Improvement (IHI). Boston: IHI. Available at: https://www.ihi.org/. Accessed April 27, 2022.

[29] Canadian Patient Safety Institute (CPSI). Ottawa: CPSI. Available at: https://www.patientsafetyinstitute.ca/. Accessed April 27, 2022.

[30] Jauregui K Liu S Patanwala A Begley D Khor KE Bugeja B Fong I Rimington J Penm J. Effectiveness of a discharge analgesia guideline on discharge opioid prescribing after a surgical procedure from a tertiary metropolitan hospital. J Opioid Manag 2024;20:329–38. 39321053 10.5055/jom.0863

[31] Kharasch ED Clark JD Adams JM. Opioids and public health: the prescription opioid ecosystem and need for improved management. Anesthesiology 2022;136:10–30. 34874401 10.1097/ALN.0000000000004065 PMC10715730

[32] Lawrence M Olesen F. Indicators of quality in health care. Eur J Gen Pract 1997;3:103–8.

[33] Levy N Mills P. Controlled-release opioids cause harm and should be avoided in management of postoperative pain in opioid naïve patients. Br J Anaesth 2019;122:e86–90. 30915981 10.1016/j.bja.2018.09.005

[34] Liu S Athar A Quach D Patanwala AE Naylor JM Stevens JA Levy N Knaggs RD Lobo DN Penm J. Risks and benefits of oral modified-release compared with oral immediate-release opioid use after surgery: a systematic review and meta-analysis. Anaesthesia 2023;78:1225–36. 37415284 10.1111/anae.16085 PMC10952256

[35] Liu S Gnjidic D Nguyen J Penm J. Effectiveness of interventions on the appropriate use of opioids for noncancer pain among hospital inpatients: a systematic review. Br J Clin Pharmacol 2020;86:210–43. 31863503 10.1111/bcp.14203 PMC7015758

[36] Liu S Patanwala AE Naylor JM Levy N Knaggs R Stevens JA Bugeja B Begley D Khor KE Lau E Allen R Adie S Penm J. Impact of modified-release opioid use on clinical outcomes following total hip and knee arthroplasty: a propensity score-matched cohort study. Anaesthesia 2023;78:1237–48. 37365700 10.1111/anae.16070 PMC10952779

[37] MacKay DR Stader D. Colorado program significantly reduces opioid prescribing in 10 EDs during six-month period. Management 2018;30:1–6.

[38] Grey Matters. Ottawa: Canadian Agency for Drugs and Technologies in Health. Available at: https://www.cadth.ca/grey-matters. Accessed April 27, 2022.

[39] O’McCracken M. Endnote X9. Philadelphia: Clarivate, 2022.

[40] World Health Organisation (WHO). Opioid Overdose. Geneva: WHO; https://www.who.int/news-room/fact-sheets/detail/opioid-overdose#:˜:text=Due%20to%20their%20pharmacological%20effects,deaths%20caused%20by%20opioid%20overdose. (2021, Accessed April 27, 2022).

[41] Page MJ McKenzie JE Bossuyt PM Boutron I Hoffmann TC Mulrow CD Shamseer L Tetzlaff JM Akl EA Brennan SE Chou R Glanville J Grimshaw JM Hróbjartsson A Lalu MM Li T Loder EW Mayo-Wilson E McDonald S McGuinness LA Stewart LA Thomas J Tricco AC Welch VA Whiting P Moher D. The PRISMA 2020 statement: an updated guideline for reporting systematic reviews. BMJ 2021;372:n71. 33782057 10.1136/bmj.n71 PMC8005924

[42] Penm J MacKinnon NJ Connelly C Mashni R Lyons MS Hooker EA Winstanley EL Carlton-Ford S Tolle E Boone J Koechlin K Defiore-Hyrmer J. Emergency physicians' perception of barriers and facilitators for adopting an opioid prescribing guideline in Ohio: a qualitative interview study. J Emerg Med 2019;56:15–22. 30342861 10.1016/j.jemermed.2018.09.005 PMC6549497

[43] Penm J MacKinnon NJ Mashni R Lyons MS Hooker EA Winstanley EL Carlton-Ford S Connelly C Tolle E Boone J Koechlin K Defiore-Hyrmer J. Statewide cross-sectional survey of emergency departments' adoption and implementation of the Ohio opioid prescribing guidelines and opioid prescribing practices. BMJ Open 2018;8:e020477. 10.1136/bmjopen-2017-020477 PMC6042556 29961010

[44] Phinn K Liu S Patanwala AE Penm J. Effectiveness of organizational interventions on appropriate opioid prescribing for noncancer pain upon hospital discharge: a systematic review. Br J Clin Pharmacol 2023;89:982–1002. 36495313 10.1111/bcp.15633

[45] Agency for Healthcare Research and Quality (AHRQ). Rockville: AHRQ. Available at: https://www.ahrq.gov/. Accessed April 27, 2022.

[46] Rizk E Swan JT Cheon O Colavecchia AC Bui LN Kash BA Chokshi SP Chen H Johnson ML Liebl MG Fink E. Quality indicators to measure the effect of opioid stewardship interventions in hospital and emergency department settings. Am J Health Syst Pharm 2019;76:225–35. 30715186 10.1093/ajhp/zxy042

[47] Samuels EA D'Onofrio G Huntley K Levin S Schuur JD Bart G Hawk K Tai B Campbell CI Venkatesh AK. A quality framework for emergency department treatment of opioid use disorder. Ann Emerg Med 2019;73:237–47. 30318376 10.1016/j.annemergmed.2018.08.439 PMC6817947

[48] Schirle L Stone AL Morris MC Osmundson SS Walker PD Dietrich MS Bruehl S. Leftover opioids following adult surgical procedures: a systematic review and meta-analysis. Syst Rev 2020;9:139. 32527307 10.1186/s13643-020-01393-8 PMC7291535

[49] National Health Service (NHS). London: NHS. Available at: https://www.nhs.uk/. Accessed April 27, 2022.

[50] Shrestha S Khatiwada AP Sapkota B Sapkota S Poudel P Kc B Teoh SL Blebil AQ Paudyal V. What is “opioid stewardship?” An overview of current definitions and proposal for a universally acceptable definition. J Pain Res 2023;16:383–94. 36798077 10.2147/JPR.S389358 PMC9926985

[51] Stanley B Norman AF Collins LJ Zographos GA Lloyd-Jones DM Bonomo A Bonomo YA. Opioid prescribing in orthopaedic and neurosurgical specialties in a tertiary hospital: a retrospective audit of hospital discharge data. ANZ J Surg 2018;88:1187–92. 30306703 10.1111/ans.14873

[52] Sterne JA Hernán MA Reeves BC Savović J Berkman ND Viswanathan M Henry D Altman DG Ansari MT Boutron I Carpenter JR Chan AW Churchill R Deeks JJ Hróbjartsson A Kirkham J Jüni P Loke YK Pigott TD Ramsay CR Regidor D Rothstein HR Sandhu L Santaguida PL Schünemann HJ Shea B Shrier I Tugwell P Turner L Valentine JC Waddington H Waters E Wells GA Whiting PF Higgins JP. ROBINS-I: a tool for assessing risk of bias in non-randomised studies of interventions. BMJ 2016;355:i4919. 27733354 10.1136/bmj.i4919 PMC5062054

[53] Suckling B Pattullo C Liu S James P Donovan P Patanwala A Penm J. Persistent opioid use after hospital discharge in Australia: a systematic review. Aust Health Rev 2022;46:367–80. 35545810 10.1071/AH21353

[54] Tay HP Wang X Narayan SW Penm J Patanwala AE. Persistent postoperative opioid use after total hip or knee arthroplasty: a systematic review and meta-analysis. Am J Health Syst Pharm 2022;79:147–64. 34537828 10.1093/ajhp/zxab367 PMC8513405

[55] Terrell KM Hustey FM Hwang U Gerson LW Wenger NS Miller DK, Society for Academic Emergency Medicine SAEM Geriatric Task Force. Quality indicators for geriatric emergency care. Acad Emerg Med 2009;16:441–9. 19344452 10.1111/j.1553-2712.2009.00382.x

[56] Thomas SK McDowell SE Hodson J Nwulu U Howard RL Avery AJ Slee A Coleman JJ. Developing consensus on hospital prescribing indicators of potential harms amenable to decision support. Br J Clin Pharmacol 2013;76:797–809. 23362926 10.1111/bcp.12087 PMC3853538

[57] Tyndall J. The AACODS checklist is designed to enable evaluation and critical appraisal of grey literature. Chin J Evid Based Med 2010;7:507–13.

[58] Australian Institute of Health and Welfare (AIHW). Opioid harm in Australia: And comparisons between Australia and Canada. Canberra: AIHW; 2018.

[59] Australian Institute of Health and Welfare (AIHW). National opioid pharmacotherapy statistics annual data collection. Canberra: AIHW; https://www.aihw.gov.au/reports/alcohol-other-drug-treatment-services/national-opioid-pharmacotherapy-statistics/contents/summary (2021, Accessed April 27, 2022).

[60] Winstanley EL Zhang Y Mashni R Schnee S Penm J Boone J McNamee C MacKinnon NJ. Mandatory review of a prescription drug monitoring program and impact on opioid and benzodiazepine dispensing. Drug Alcohol Depend 2018;188:169–74. 29778769 10.1016/j.drugalcdep.2018.03.036 PMC6528173

