# Supplementary material for: Quality indicators for opioid stewardship interventions in hospital inpatient and emergency departments: a systematic review
Source: Pain Rep. 2025 Jun 18;10(4):e1284. doi: 10.1097/PR9.0000000000001284 (PMC12178301; doi:10.1097/PR9.0000000000001284)
Supplement: SUPPLEMENTARY MATERIAL [file painreports-10-e1284-s001.pdf]

APPENDIX 1: **Search Strategy for Electronic Databases**

OVID Medline

1 exp Analgesics, Opioid/
2 exp Narcotics/
3 (acetyldihydrocodeine or alfentanil or allylprodine or alphamethylfentanyl or alphaprodine or benzylmorphine or betaprodine or buprenorphine or butorphanol or bremazocine or codeine or contin or dextromoramide or dextropropoxyphene or dezocine or diacetylmorphine or diamorphine or dihydrocodeine or dihydromorphine or dihydromorphone or diphenoxylate or dipipanone or enadoline or ethylketazocine or ethylmorphine or etonitazene or etorphine or fentanyl or heroin or hydrocodone or hydromorphin* or hydromorphone or ketazocine or ketobemidone or lefetamine or levomethadon or levomethadyl or levomethorphan* or levorphanol or loperamide or meperidine or meptazinol or methadone or methadyl or methylmorphine or morphin* or nalbuphine or narcotic* or nicocodeine or nicomorphine or normorphine or noscapin* or ohmefentanyl or opiate* or opioid* or opium or oripavine or oxycodone or oxycontin or oxymorphone or papaveretum or papaverin or pentazocine or percocet or peronine or pethidine or phenazocine or phencyclidine or pholcodine or piritramid* or prodine or promedol or propoxyphene or remifentanil or sufentanil or tapentadol or thebaine or tilidine).mp.
4 1 or 2 or 3
5 Inpatients/
6 (inpatient* adj2 hospital).mp.
7 (inpatient* adj2 setting*).mp.
8 hospital.mp. or Hospitals/ or (hospital* adj2 setting*).mp.
9 exp Hospital Departments/
10 (acute adj2 care).mp. or acute disease/
11 emergency service, hospital/ or trauma centers/ or (emergency adj2 department).mp.
12 5 or 6 or 7 or 8 or 9 or 10 or 11
13 (intervention* or recommend* or guideline* or appropriate prescrib* or stewardship* or program* or (intervention* adj6 (improve* or under-prescrib* or under prescrib* or over-prescrib* or over prescrib* or impact* or prescrib*))).mp.
14 exp Quality Assurance, Health Care/ or exp Quality Indicators, Health Care/ or "Quality of Health Care"/
15 (quality indicator* or quality measure* or performance indicator* or performance measure* or quality improvement* or improvement measure*).mp.
16 14 or 15
17 4 and 12 and 13 and 16

APPENDIX 2

Quality assessment summary of included studies using a consensus-based or Delphi method using the *COSMIN Risk of bias tool*[36]

**Study Authors**	**Allen et al.[41]**	
**Design requirements**	**Judgement**	**Reason for judgement**
Were patients stable in the time between the repeated measurements on the construct to be measured?	Inadequate	Only one group of participants remained consistent throughout the iterative rounds
Was the time interval between the repeated measurements appropriate?	Doubtful	Time interval not stated
Were the measurement conditions similar for the repeated measurements - except for the condition being evaluation as a source of variation?	Very good	Measurement process remained consistent
Did the professional(s) administer the measurement without knowledge of scores or values of other repeated measurement(s) in the same patients?	Doubtful	Unclear
Did the professional(s) assign scores or determine values without knowledge of the scores or values of other repeated measurement(s) in the same patients?	Inadequate	The results of previous rounds were shared with participants
Were there any other important flaws in the design or statistical methods of the study?	Very good	No
**Overall judgement**	Inadequate	

**Study Authors**	**Fox et al.[42]**	
**Design requirements**	Judgement	Reason for judgement
Were patients stable in the time between the repeated measurements on the construct to be measured?	Very good	All originally included participants completed all rounds of evaluation
Was the time interval between the repeated measurements appropriate?	Doubtful	No time interval state
Were the measurement conditions similar for the repeated measurements - except for the condition being evaluation as a source of variation?	Very good	Measurement conditions were the same
Did the professional(s) administer the measurement without knowledge of scores or values of other repeated measurement(s) in the same patients?	Very good	Participants individually evaluated the quality indicators
Did the professional(s) assign scores or determine values without knowledge of the scores or values of	Inadequate	The results of previous rounds were shared with participants

other repeated measurement(s) in the same patients?		
Were there any other important flaws in the design or statistical methods of the study?	No	
**Overall judgement**	Inadequate	

**Study Authors**	**Rizk et al.[38]**	
**Design requirements**	Judgement	Reason for judgement
Were patients stable in the time between the repeated measurements on the construct to be measured?	Inadequate	Participants declined between the first, second, and third round respectively
Was the time interval between the repeated measurements appropriate?	Doubtful	Time interval not stated
Were the measurement conditions similar for the repeated measurements - except for the condition being evaluation as a source of variation?	Inadequate	Measurement conditions included online surveys as well as panel meetings
Did the professional(s) administer the measurement without knowledge of scores or values of other repeated measurement(s) in the same patients?	Inadequate	The panel meetings included face-to-face discussion of values
Did the professional(s) assign scores or determine values without knowledge of the scores or values of other repeated measurement(s) in the same patients?	Inadequate	The results of previous rounds were shared with participants
Were there any other important flaws in the design or statistical methods of the study?	No	
**Overall judgement**	Inadequate	

**Study Authors**	**Samuels et al.[39]**	
**Design requirements**	Judgement	Reason for judgement
Were patients stable in the time between the repeated measurements on the construct to be measured?	Doubtful	Unclear
Was the time interval between the repeated measurements appropriate?	Doubtful	Time interval not stated
Were the measurement conditions similar for the repeated measurements - except for the condition being evaluation as a source of variation?	NA	
Did the professional(s) administer the measurement without knowledge of scores or values of other repeated measurement(s) in the same patients?	Inadequate	Results were obtained through group discussion

Did the professional(s) assign scores or determine values without knowledge of the scores or values of other repeated measurement(s) in the same patients?	Doubtful	Unclear
Were there any other important flaws in the design or statistical methods of the study?	Method unclear	
**Overall judgement**	Inadequate	

**Study Authors**	**Terrell et al.[40]**	
**Design requirements**	Judgement	Reason for judgement
Were patients stable in the time between the repeated measurements on the construct to be measured?	Inadequate	A completely different set of participants was used for each round of evaluation
Was the time interval between the repeated measurements appropriate?	Doubtful	Time interval not stated
Were the measurement conditions similar for the repeated measurements - except for the condition being evaluation as a source of variation?	Inadequate	Some measurements were independent, others were by group discussion
Did the professional(s) administer the measurement without knowledge of scores or values of other repeated measurement(s) in the same patients?	Inadequate	Group discussion
Did the professional(s) assign scores or determine values without knowledge of the scores or values of other repeated measurement(s) in the same patients?	Inadequate	The results of previous rounds were shared with participants
Were there any other important flaws in the design or statistical methods of the study?	No	
**Overall judgement**	Inadequate	

**Study Authors**	**Thomas et al.[43]**	
**Design requirements**	Judgement	Reason for judgement
Were patients stable in the time between the repeated measurements on the construct to be measured?	Very good	Participants remained the same in each round
Was the time interval between the repeated measurements appropriate?	Doubtful	Time interval not stated
Were the measurement conditions similar for the repeated measurements - except for the condition being evaluation as a source of variation?	Very good	Likert scale used throughout

Did the professional(s) administer the measurement without knowledge of scores or values of other repeated measurement(s) in the same patients?	Very good	Review was completed independently by participants
Did the professional(s) assign scores or determine values without knowledge of the scores or values of other repeated measurement(s) in the same patients?	Inadequate	The results of previous rounds were shared with participants
Were there any other important flaws in the design or statistical methods of the study?	No	
**Overall judgement**	Inadequate	

APPENDIX 3

Quality assessment of included grey literature articles using the *Authority, Accuracy, Coverage, Objectivity, Date and Significance (AACODS) checklist*[37]

**Study Author**	**Joint Commission**[37]	
**Criteria**	**Judgement**	**Reason for judgement**
**Authority**	Yes	The organisation is reputable and responsible for hospital accreditation internationally. There is a detailed reference list.
**Accuracy**	Yes	Sourced by appropriate references, methodology, and representative of work in the field.
**Coverage**	No	Limits to content coverage have not been stated adequately.
**Objectivity**	Yes	Work balanced in presentation
**Date**	Yes	Date has been stated
**Significance**	Yes	Provides standards for accreditation, integral to hospital practice.
**Overall judgement**	Low risk of bias	
**Study Author**	**Australian Commission on Safety and Quality in Health Care**[21]	
**Criteria**	**Judgement**	**Reason for judgement**
**Authority**	Yes	The organisation is reputable. There is a detailed reference list.
**Accuracy**	Yes	Sourced by appropriate references, peer-reviewed, and representative of work in the field.
**Coverage**	Yes	Limits of coverage are clearly stated.
**Objectivity**	Yes	Work balanced in presentation
**Date**	Yes	Date has been stated
**Significance**	Yes	Provides standards for accreditation, integral to hospital practice.
**Overall judgement**	Low risk of bias	
